# Kisspeptin Protein in Seminal Plasma Is Positively Associated with Semen Quality: Results from the MARHCS Study in Chongqing, China

**DOI:** 10.1155/2019/5129263

**Published:** 2019-01-09

**Authors:** Peng Zou, Xiaogang Wang, Qing Chen, Huan Yang, Niya Zhou, Lei Sun, Hongqiang Chen, Jinyi Liu, Lin Ao, Zhihong Cui, Jia Cao

**Affiliations:** ^1^Key Lab of Medical Protection for Electromagnetic Radiation, Ministry of Education of China, Institute of Toxicology, College of Preventive Medicine, Third Military Medical University, Chongqing 400038, China; ^2^Frontier Defence Medical Service Training Group, Army Medical University, Hutubi 831200, China; ^3^College of Pharmaceutical Sciences, Southwest University, Chongqing 400715, China

## Abstract

**Objectives:**

To study the associations between kisspeptin levels in seminal plasma and blood plasma and semen quality.

**Materials and Methods:**

We conducted a male reproductive health survey in June 2014. A total of 666 volunteers were recruited from colleges in Chongqing, China. All volunteers completed a questionnaire including information on domestic characteristics and some potential confounders. We tested the kisspeptin levels in both blood and seminal plasma. Total seminal kisspeptin was calculated as the concentration of kisspeptin in seminal plasma multiplied by semen volume. Semen samples were tested according to the 2010 World Health Organization's (WHO) guidelines. Spearman correlation and multivariate linear regression were used to explore the association between kisspeptin concentrations in seminal plasma and blood plasma and semen quality. Potential confounders that were adjusted for included age, abstinence time, body mass index (BMI), grade, and smoking.

**Results:**

The median of kisspeptin levels in seminal plasma was 60,000 times higher than kisspeptin in blood plasma (28.0 × 10^6^ pg/ml versus 448.9 pg/ml). Each interquartile range (IQR) of kisspeptin in seminal plasma was associated with a 4.6% (95% confidence interval [CI]: 1.6%–7.6%) increase in sperm concentration. Each IQR of total kisspeptin was associated with a 7.7% (95% CI: 4.4%–11.0%) increase in total sperm number and a 7.8% (95% CI: 4.0%–11.7%) increase in total motile sperm count. Kisspeptin levels were further classified into quartiles and Q1 was set as the reference level. Subjects in the high total kisspeptin group had 57.5% (95% CI: 33.2%–86.2%) higher total sperm number than the reference group.

**Conclusion:**

The positive association between kisspeptin levels in seminal plasma and semen quality supported an important role for the* KISS1/GPR54 *system in male reproductive health. Kisspeptin may be a potential marker of male reproductive health and an alternative strategy for treating infertility.

## 1. Introduction

Routine semen analysis is a widely used clinical application recommended by the World Health Organization (WHO). However, the power of semen analysis for the evaluation of male fertility is limited [1]. Milardi et al. highlighted the possibility of spontaneous conception with semen parameters below WHO reference values [2]. Some people with normal routine semen parameters can still be infertile [3]. Male fertility is maintained by a series of biological processes such as spermatogenesis, sperm maturation, and sperm transport [4]. During these processes, seminal plasma serves as a survival medium that delivers sperm to fertilize the oocyte. Seminal plasma is a kind of mixed fluid produced from testes, epididymis, and accessory sex glands [5]. Seminal plasma constituents exert various biological functions, such as sperm function, fertilization, and even embryo implantation and development [6]. Increased knowledge of seminal components can enable us to identify novel biomarkers of fertility [7].

Kisspeptins are a family of peptides encoded by the* KISS1* gene [8]. The kisspeptin precursor consists of 145 amino acids, and it is cleaved to different peptides such as kp-54, -14, -13, and -10 [9]. All of these cleaved peptides are sufficient to activate the G protein-coupled receptor, GPR54 [9]. The* KISS1/GPR54* system was found to be expressed in several tissues such as the placenta, small intestine, hypothalamus, and testes [10]. The* KISS1/GRP54* system plays a key role in the initiation of the Hypothalamic–Pituitary–Gonadal (HPG) axis, reproductive hormone secretion, puberty onset, fertility maintenance, and even metabolism [11]. In addition, Hsu et al. [12] reported the expression of GPR54 has been found in mouse spermatids and mature spermatozoa. Another study showed that both kisspeptin and GPR54 were expressed in human sperm [13], thereby indicating the potential involvement of the* KISS1/GPR54* system in fertility regulation. In a study including 176 men in the clinic, serum kisspeptin were significantly lower in infertile men [14]. To date, the* KISS1/GPR54* system has been recognized as a crucial regulator in the male reproductive system.

The origin and function of the* KISS1/GPR54* system are not yet completely clear. The aim of this study was to determine whether kisspeptin is present in seminal plasma. If it exists, the associations between semen quality and kisspeptin could be identified.

## 2. Methods

### 2.1. Study Design and Population

This survey was based on the first follow-up survey of Male Reproductive Health in Chongqing College Students (MARHCS) and was conducted in June 2014. Male volunteers were recruited from the university town in Chongqing China. Semen and blood samples were obtained. Subjects completed a questionnaire regarding demographic characteristics, medical history, and lifestyle, and so on. Potential confounders such as age and abstinence time were also included in the questionnaire. Physical examinations were conducted by an experienced urologist. Any urogenital abnormalities such as varicocele were recorded. Height and weight were measured in this step, and body mass index (BMI) was defined as kg/m^2^. Volunteers who met the following criteria were excluded from the baseline study: (1) age < 18 years old; (2) abstinence time < 2 days or > 7 days; (3) a history of any urogenital diseases; and/or (4) any urogenital abnormalities detected by an urologist at the physical examination stage. In the end, a total of 666 subjects were eligible for this survey.

This study was approved by the Ethics Committees of Third Military Medical University. Informed consent was obtained from all subjects.

### 2.2. Semen Analysis

Subjects were instructed to keep abstinence time between 2 and 7 days before participating in the survey. Semen samples were collected by masturbation in a private room, and subsequently incubated at 37°C for liquefaction. Semen volume was determined by weighting assuming 1g=1ml. Smears for morphology were measured using Diff-Quick staining (Bred life science, China, Product Code: BRED-015). Sperm concentration and motility were measured by computer-aided sperm analysis (SCA CASA System; Microptic S.L., Barcelona, Spain). The detailed procedure was in accordance with the instruction of the analysis system. Total motility equals progressive motility plus nonprogressive motility. Total sperm number = semen volume × sperm concentration. Total motile sperm count = total sperm number × total motility. In order to reduce the variation in assessment of semen characteristics, all semen samples were analyzed by one well-trained technician throughout the survey, who was well trained in semen analysis under the guidance of the Chongqing Science and Technology Commission.

### 2.3. Kisspeptin Measurement

Blood sample was centrifuged at 1,000 g for 10 min at room temperature. Plasma was separated and stored at −80°C before the assay was done. Plasma kisspeptin was measured by enzyme-linked immunosorbent assay (ELISA) (Cloud-clone, USA) without dilution. The detection ranges from 31.2 to 2000 pg/ml. The coefficients of variation for intra and interassays were <10% and <10%, respectively. Semen samples were centrifuged at 300 g for 7 min at room temperature. The supernatant seminal plasma was removed and saved at −80°C before the assay was done. Kisspeptin in seminal plasma was measured by enzyme-linked immunosorbent assay (ELISA) kits (Cusabio, Wuhan, China) with dilution. Most of the seminal plasma samples were diluted at a ratio of 1:20,000 by serial dilutions. If samples generate values higher than the highest standard, they will be diluted at a higher ratio and measured again. The measurement was in accordance with the manufacturer's instruction. The detection ranges from 0.312 to 20 ng/ml. The coefficients of variation for intra and interassays were <8% and <10%, respectively. Total kisspeptin = kisspeptin in seminal plasma× semen volume.

### 2.4. Statistical Analyses

For demographic characteristics, we calculated the median (25th and 75th percentile) of continuous variables and percentages of categorical variables. Kisspeptin levels were evaluated using histograms. We explored the associations between kisspeptin and semen parameters using Spearman correlation.

We used multivariate linear regression models in order to investigate the associations between kisspeptin in seminal plasma, total kisspeptin, and semen parameters. Potential confounders were selected according to previously published articles [15]. We adjusted age (years), BMI grade (<18.5, 18.5–23.9, 24–27.9, >28 kg/m^2^), abstinence time (days), and smoking (never, ever, current) in the regression models using a stepwise method. Season and temperature, considered to be possible confounders, were not adjusted for because this survey was conducted over a short period in June. Because sperm concentration, semen volume, and total sperm number were in skewed distribution, they were log-transformed to achieve the normal distribution of the residuals. Beta coefficients were back transformed as % change in semen parameters per interquartile range (IQR) increase in kisspeptin levels. Furthermore, kisspeptin levels were classified into four groups according to the quartiles (Q1 to Q4, from the lowest to the highest). We calculated the % change of Q2–4, with Q1 as the reference level.

A *P* value < 0.05 was considered significant. All statistical analyses were performed using SPSS 18.0 (IBM).

## 3. Results

### 3.1. Subjects' Demographic Characteristics, Semen Quality, and Kisspeptin Levels

The demographic characteristics were presented as median (25th and 75th percentile) or percentages (Table 1). The ages of the subjects ranged from 19 to 27 years old. In addition, 75.3% of the subjects had normal weight, which is defined as a BMI ranging between 18.5 and 23.9 kg/m^2^. Semen parameters were dichotomized according to the WHO guideline criteria. The normal rates of semen volume, sperm concentration, total sperm number, progressive motility, total motility, sperm morphology, and total motile sperm count were 98.2%, 95.1%, 96.2%, 91.6%, 99.5%, 91.7%, and 96.3%, respectively. The median of kisspeptin in seminal plasma was 60,000 times higher than that in blood plasma (28 × 10^6^ pg/ml versus 448.9 pg/ml) (Table 1).

### 3.2. Distribution of Kisspeptin Levels and Their Associations with Semen Parameters

**Figure 1:**
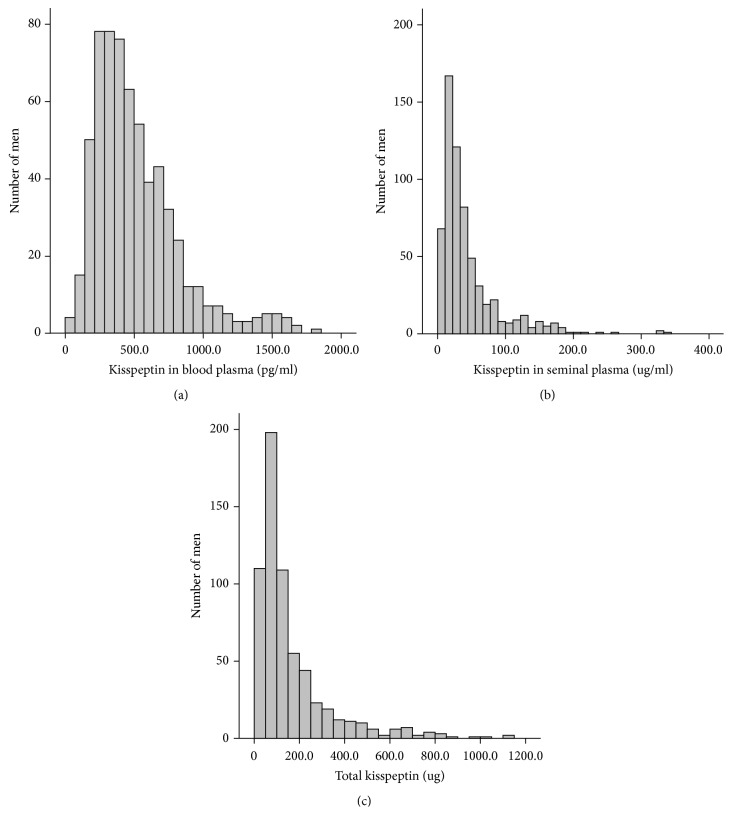
The distributions of kisspeptin in blood plasma (a), kisspeptin in seminal plasma (b), and total kisspeptin (c).

The distributions of kisspeptin in blood plasma and seminal plasma and total kisspeptin are shown in Figure 1. Spearman's correlation coefficient between kisspeptin in blood plasma and kisspeptin in seminal plasma was –0.056 (*P*=0.171) (Table 2). Kisspeptin in seminal plasma was associated with sperm concentration and semen volume (r=0.124 and –0.104,* P*=0.002 and* P*=0.009, respectively). Total kisspeptin was associated with semen volume (r=0.332,* P*≤0.001) (Table 2). In addition, both total sperm number and total motile sperm count were associated with total kisspeptin (r=0.230 and 0.194,* P*≤0.001 and* P*≤0.001, respectively) (Table 2). The Spearman correlation analyses indicated that BMI grade was negatively associated with sperm concentration, semen volume and total sperm number (r=–0.082, –0.105, and –0.137;* P*=0.038, 0.007, and <0.001, respectively). No significant association was observed between kisspeptin and BMI (*P* >0.05, data not shown). No statistically significant associations were found between kisspeptin and reproductive hormones (P >0.05, data not shown).

### 3.3. Associations between Kisspeptin in Seminal Plasma and Semen Parameters

We used multivariate linear regression models to explore the associations between kisspeptin and semen parameters. Potential confounders that were adjusted included age, abstinence time, BMI grade, and smoking. Sperm concentration was positively associated with per IQR increase in kisspeptin in seminal plasma (% change=4.6%,* P*=0.003) (Table 3). Both kisspeptin in seminal plasma and total kisspeptin were positively associated with total sperm number. Each IQR increase in kisspeptin in seminal plasma and total kisspeptin were associated with a 3.3% and 7.7% (*P*=0.047 and* P*≤0.001, respectively) increase in total sperm number (Table 3). In addition, each IQR increase in total kisspeptin was associated with a 7.8% increase in total motile sperm count (*P*≤0.001) (Table 3).

**Figure 2:**
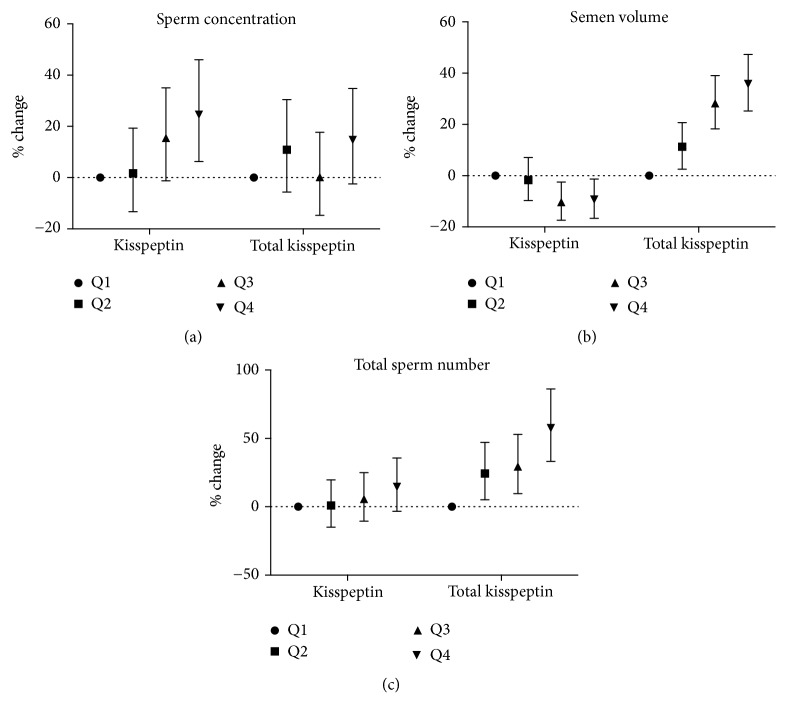
Associations of categorized kisspeptin in seminal plasma and categorized total kisspeptin with sperm concentration (a), total sperm number (b), and total motile sperm count (c). Kisspeptin levels were classified into four groups according to the quartiles (Q1 to Q4, from the lowest to the highest). We set the Q1 group as the reference level.

We conducted further analyses when kisspeptin in seminal plasma and total kisspeptin were categorized into four groups according to their percentiles. The percentiles for kisspeptin in seminal plasma were 18.0, 28.0, and 52.0 ug/ml. The percentiles for total kisspeptin were 60.0, 101.3, and 196.8 ug. Using Q1 (lowest kisspeptin group) as the reference, it was seen that subjects in the Q4 group had a higher sperm concentration (24.6%, 95% CI: 6.3%– 46.0%) (Figure 2(a)). Total sperm number was positively associated with total kisspeptin (*P*≤0.001). Subjects in the Q3 and Q4 groups had a higher total sperm number (% change=29.4% and 57.5%,* P*=0.003 and* P*≤0.001, respectively) (Figure 2(b)). In addition, total motile sperm count was positively associated with total kisspeptin (*P*≤0.001). Subjects in the Q3 and Q4 groups had higher total motile sperm counts (% change=26.5% and 56.7%;* P*=0.017 and* P *≤0.001, respectively) (Figure 2(c)).

## 4. Discussion

In this cross-sectional study including 666 young male volunteers in Chongqing China, we found that kisspeptin is present in human seminal plasma. This was the first study reporting that total kisspeptin was positively associated with sperm concentration, total sperm number, and total motile sperm count.

In the present study, the median value of kisspeptin in seminal plasma was 28 ug/ml, which was about 60,000 times higher than kisspeptin in blood plasma. Some research has indicated that testes may be an important source of kisspeptin in the blood. Mouse gonadectomy led to the intense decrease of serum kisspeptin [16]. In our study, the correlation between kisspeptin in blood plasma and seminal plasma was not statistically significant different (Supplemental figure (available here)). This may due to the big difference between kisspeptin in seminal plasma and kisspeptin in blood plasma. Furthermore, the possibility exists that the testicular origins of kisspeptin in blood and in seminal plasma were different. In addition, we further compared the kisspeptin level in blood plasma with findings from other studies. The median of plasma kisspeptin in our study was 448.9 pg/ml. Different studies have reported kisspeptin levels at 6.2 ng/ml [17], 0.6 ng/ml [18], and 2.9 ng/ml [19]. Such differences may be attributed to the age of the subjects in the various studies. It has been reported that the kisspeptin level in blood plasma was highly correlated with age but the association with age was throughout puberty, rather than in adulthood. Indeed, it was significantly higher in children than in adults [19].

Based on the positive association between kisspeptin and total sperm number in addition to total motile sperm count, it was hypothesized that kisspeptin may accelerate the process of spermatogenesis. Various supportive evidence derived from animals exists. In the seminiferous tubule of adult mice, kisspeptin and GPR54 were both detected in the elongated spermatids, but not in spermatozoa or spermatocytes [12]. Thus, the appearance of the* KISS1/GPR54* system accompanies spermatogenesis. In another study conducted in sexually immature adult chum mackerel, Selvaraj et al. [20] reported that the subcutaneous administration of kisspeptin led to the acceleration of spermatogenesis. Furthermore, Chianese et al. [21] cultured the testes of anuran amphibian* Pelophylax esculentu*s in its reproductive period and found that the treatment with kisspeptin accelerated the germ cell progression. However, the evidence was not conclusive. The picture is still somewhat complicated. The previously cited studies reported the acceleration of spermatogenesis due to kisspeptin, but some studies showed an inhibitory effect. Researchers found that kisspeptin treatment for 30 days led to the testicular degeneration in male rats, including decreased testicular weight, degeneration of the seminiferous tubules, and decreases in inhibin B and testosterone [22]. This might be due to the desensitization of the HPG axis caused by chronic kisspeptin administration. Another study showed that the testicular degeneration in male rats occurred only after 12 h of kisspeptin administration [23]. Similarly, kisspeptin administration induced degeneration in male rat testes. The amount of elongated spermatid, preleptotene spermatocytes, and daily sperm production decreased significantly after kisspeptin treatment [24]. Whether the effects of kisspeptin on spermatogenesis were direct or indirect is still unclear. First, subcutaneous administration of kisspeptin led to a decrease in testosterone [22]. Tompson et al. indicated that kisspeptin-induced testicular degeneration appears to be mediated by the HPG axis [23]. On the other hand, both kiss1 and its receptor GPR54 are expressed in the testes [25]. The possibility of a direct role of kisspeptin on spermatogenesis cannot be excluded. In addition, we found a negative association between semen volume and kisspeptin in seminal plasma. There is a possibility that the association between semen volume and kisspeptin might reflect an impairment in accessory gland function. In order to test this hypothesis, we need to examine the kisspeptin level of volunteers with accessory gland abnormality. Also, we found that BMI grade was negatively associated with semen quality. This result was consistent with other studies (Sermondade et al. 2013). Thus, BMI was adjusted for in the regression models.

We found no association between blood plasma kisspeptin and semen quality. Although one study reported that serum kisspeptin in infertile men was lower than kisspeptin in fertile men, the mean of serum kisspeptin in fertile men was 23.3 ng/ml compared to < 10.0 ng/ml in different infertile men (such as azoospermic, asthenozoospermic, and oligozoospermic). The associations between blood plasma kisspeptin and semen quality needs to be validated in the future. Thus, the level of kisspeptin is involved in many biological processes. We preferred to hypothesize that kisspeptin in seminal plasma is more likely to be associated with reproduction.

Both kisspeptin and GPR54 were expressed in human testes [26]. The enrichment of kisspeptin reflected the underlying function in fertility. The* KISS1/GPR54* system may be involved in the regulation of sperm chemotaxis, which has been demonstrated in humans [27]. Sperms move toward a certain direction because of the gradient concentration of attractants. Attractants derived from follicular fluids or cumulus cells induce the turning movement of sperms. Otherwise, sperms exhibit a linear movement [27]. Calcium ion (Ca^2+^) is an important factor that regulates the behavior of sperm chemotaxis. Exposure to sperm attractant induces [Ca^2+^] fluctuations [28]. The mammalian sperm chemotaxis can be mediated by G-protein-coupled receptors such as odorant receptors [29]. Ca^2+^ fluctuations and G-protein-coupled receptors-were both present in the* KISS1/GPR54* system [13, 30]. Therefore, regulation of sperm chemotaxis is a possible function of the* KISS1/GPR54* system.

There were several strengths in this study. First, this study was based on the first follow-up survey of a cohort study (MARHCS). It was established in June 2013 and followed up in June 2014. The volunteers' similar background, including the narrow age range, the same educational background, and a relatively fixed living area, may have reduced some unpredicted factors that would have biased our results. Second, the volunteers were recruited from the general population, not from the clinic, and the sample size was relatively large. Third, the kisspeptin level in seminal plasma has never been reported. We revealed high level seminal plasma kisspeptin levels and made a preliminary exploration of its function.

There were also several limitations in this study. First, this was a cross-sectional study, although the data were from the first follow-up survey of a cohort. Therefore, reverse causation cannot be excluded. There is a possibility that cross-talk between sperm and epididymis or ductus deferens could regulate kisspeptin expression. Second, only one semen sample was obtained from each volunteer. Third, subjects' paternity statuses were very helpful to provide some novel information that really impact clinical practice, but all subjects in this study were college students, and none of them had any children. Last, semen analysis was performed by the CASA method, which is not a standardized method according to the manual of WHO.

In conclusion, we revealed kisspeptin expression in seminal plasma, and we found that total kisspeptin was positively associated with total sperm number and total motile sperm count. This study provided an additional foundation for future studies on this topic. With further research exploring the association between kisspeptin and fertility, kisspeptin might be developed as a marker of fertility.

## Figures and Tables

**Table 1:** Subjects' demographic characteristics, semen parameters, and kisspeptin levels.

Characteristics	N	Value^1^
Age, y	666	21 (21, 22)
Abstinence time, days	665	4 (3, 5)
BMI grade, kg/m^2^		
<18.5	53	8.0%
18.5-23.9	498	75.3%
24.0-27.9	90	13.6%
>28	20	3.0%
Smoke		
Never	484	72.7%
Ever	35	5.3%
Current	147	22.1%
Sperm concentration, 10^6^/ml	655	52.0 (33.2, 81.7)
Semen volume, ml	655	3.5 (2.7, 4.6)
Total sperm number, 10^6^	655	193.3 (110.6, 311.0)
Progressive motility, %	655	57.0 (44.8, 67.7)
Total motility, %	655	89.4 (80.6, 94.8)
Total motile sperm count, 10^6^	655	109.82 (52.8, 184.4)
Sperm Morphology, %	652	10.5 (7.0, 15.7)
Kisspeptin A, pg/ml	626	448.9 (296.0, 668.6)
Kisspeptin B, ug/ml	631	28.0 (18.0, 52.0)
Total kisspeptin, ug	626	101.3 (60.0, 196.8)

^1^Presented as median (25th, 75th percentile) or percentage.

Kisspeptin A: kisspeptin in blood plasma; Kisspeptin B: kisspeptin in seminal plasma.

**Table 2:** Spearman correlations between kisspeptin and semen parameters.

		Kisspeptin A, pg/ml	Kisspeptin B, ug/ml	Total kisspeptin, ug	Sperm concentration, 10^6^/ml	Semen volume, ml	Total sperm number, 10^6^	Progressive motility, %	Total motility, %	Total motile sperm count, 10^6^	Sperm morphology, %
Kisspeptin A, pg/ml	Coefficient	1	-0.056	-0.043	-0.060	0.010	-0.062	-0.003	-0.017	-0.064	0.062
	*P*		0.171	0.290	0.135	0.811	0.121	0.937	0.682	0.111	0.123
	N	626	596	593	618	619	619	619	619	619	616
Kisspeptin B, ug/ml	Coefficient		1	0.883^*∗∗*^	0.124^*∗∗*^	-0.104^*∗∗*^	0.066	0.006	-0.029	0.055	-0.017
	*P*			≤0.001	0.002	0.009	0.099	0.879	0.475	0.171	0.675
	N		631	626	626	627	627	627	627	627	625
Total kisspeptin, ug	Coefficient			1	0.061	0.332^*∗∗*^	0.230^*∗∗*^	-0.011	-0.048	0.194^*∗∗*^	-0.029
	*P*				0.127	≤0.001	≤0.001	0.778	0.227	≤0.001	0.474
	N			626	625	626	625	625	625	625	621

Kisspeptin A: kisspeptin in blood plasma; Kisspeptin B: kisspeptin in seminal plasma; *∗∗*: *P*<0.01.

**Table 3:** Associations between semen parameters and kisspeptin in seminal plasma.

	Sperm concentration, 10^6^/ml		Total sperm number, 10^6^		Total motile sperm count, 10^6^	
	% change (95% CI)	*P*	% change (95% CI)	*P*	% change (95% CI)	*P*
Kisspeptin in seminal plasma, ug/ml	4.6 (1.6, 7.6)	0.003	3.3 (0.0, 6.5)	0.047	3.5 (-0.1, 7.2)	0.059
Total kisspeptin, ug	2.3 (-0.9, 5.5)	0.156	7.7 (4.4, 11.0)	≤0.001	7.8 (4.0, 11.7)	≤0.001

^1^Semen parameters were log-transformed before analyses and back-transformed to obtain the % change (with 95% CI given in the brackets) of semen parameters per IQR increase in kisspeptin. Adjusted for age, abstinence time, BMI grade, and smoking.

IQR, interquartile range; CI, confidence interval.

## Data Availability

The original data used to support the findings of this study have not been made available due to the main study (MARHCS), from which this study obtained data from, still analyzing data and preparing manuscripts for publication.

## Supplementary Materials

Supplementary Materials 
**Figure: correlation between kisspeptin in semen plasma and kisspeptin in blood plasma.** There was no statistically significant association between kisspeptin in semen plasma and kisspeptin in blood plasma.

## References

[1] Wang X., Chen Q., Zou P. (2018). Sleep duration is associated with sperm chromatin integrity among young men in Chongqing, China. *Journal of Sleep Research*.

[2] Milardi D., Grande G., Sacchini D. (2012). Male fertility and reduction in semen parameters: A single tertiary-care center experience. *International Journal of Endocrinology*.

[3] Van Der Steeg J. W., Steures P., Eijkemans M. J. C. (2011). Role of semen analysis in subfertile couples. *Fertility and Sterility*.

[4] Verze P., Cai T., Lorenzetti S. (2016). The role of the prostate in male fertility, health and disease. *Nature Reviews Urology*.

[5] Robertson S. A., Sharkey D. J. (2016). Seminal fluid and fertility in women. *Fertility and Sterility*.

[6] Nederlof I., Meuleman T., van der Hoorn M. L. P., Claas F. H. J., Eikmans M. (2017). The seed to success: The role of seminal plasma in pregnancy. *Journal of Reproductive Immunology*.

[7] Milardi D., Grande G., Vincenzoni F., Castagnola M., Marana R. (2013). Proteomics of human seminal plasma: Identification of biomarker candidates for fertility and infertility and the evolution of technology. *Molecular Reproduction and Development*.

[8] West A., Vojta P. J., Welch D. R., Weissman B. E. (1998). Chromosome localization and genomic structure of the KiSS-1 metastasis suppressor gene (KISS1). *Genomics*.

[9] Pinilla L., Aguilar E., Dieguez C., Millar R. P., Tena-Sempere M. (2012). Kisspeptins and reproduction: physiological roles and regulatory mechanisms. *Physiological Reviews*.

[10] Tena-Sempere M. (2006). GPR54 and kisspeptin in reproduction. *Human Reproduction Update*.

[11] Navarro V. M., Tena-Sempere M. (2012). Neuroendocrine control by kisspeptins: Role in metabolic regulation of fertility. *Nature Reviews Endocrinology*.

[12] Hsu M.-C., Wang J.-Y., Lee Y.-J., Jong D.-S., Tsui K.-H., Chiu C.-H. (2014). Kisspeptin modulates fertilization capacity of mouse spermatozoa. *Reproduction*.

[13] Pinto F. M., Cejudo-Román A., Ravina C. G. (2012). Characterization of the kisspeptin system in human spermatozoa. *International Journal of Andrology*.

[14] Ramzan M. H., Ramzan M., Ramzan F. (2015). Insight into the serum kisspeptin levels in infertile males. *Archives of Iranian Medicine*.

[15] Chen Q., Yang H., Zhou N. (2016). Inverse U-shaped association between sleep duration and semen quality: Longitudinal observational study (MARHCS) in chongqing, China. *SLEEP*.

[16] Salehi S., Adeshina I., Chen H. (2015). Developmental and endocrine regulation of kisspeptin expression in mouse leydig cells. *Endocrinology*.

[17] Kavvasoglu S., Ozkan Z. S., Kumbak B., Simsek M., Ilhan N. (2012). Association of kisspeptin-10 levels with abortus imminens: A preliminary study. *Archives of Gynecology and Obstetrics*.

[18] Kaya A., Orbak Z., Polat H., Çayır A., Erdil A., Döneray H. (2015). Plasma kisspeptin levels in newborn infants with breast enlargement. *Journal of Clinical Research in Pediatric Endocrinology*.

[19] Jayasena C. N., Nijher G. M. K., Narayanaswamy S. (2014). Age-dependent elevations in plasma kisspeptin are observed in boys and girls when compared with adults. *Annals of Clinical Biochemistry*.

[20] Selvaraj S., Ohga H., Nyuji M. (2013). Subcutaneous administration of Kiss1 pentadecapeptide accelerates spermatogenesis in prepubertal male chub mackerel (Scomber japonicus). *Comparative Biochemistry and Physiology - A Molecular and Integrative Physiology*.

[21] Chianese R., Ciaramella V., Fasano S., Pierantoni R., Meccariello R. (2015). Kisspeptin drives germ cell progression in the anuran amphibian *Pelophylax esculentus*: a study carried out in *ex vivo* testes. *General and Comparative Endocrinology*.

[22] Thompson E. L., Murphy K. G., Patterson M. (2006). Chronic subcutaneous administration of kisspeptin-54 causes testicular degeneration in adult male rats. *American Journal of Physiology-Endocrinology and Metabolism*.

[23] Thompson E. L., Amber V., Stamp G. W. H. (2009). Kisspeptin-54 at high doses acutely induces testicular degeneration in adult male rats via central mechanisms. *British Journal of Pharmacology*.

[24] Ramzan F., Qureshi I. Z. (2011). Intraperitoneal kisspeptin-10 administration induces dose-dependent degenerative changes in maturing rat testes. *Life Sciences*.

[25] Mei H., Doran J., Kyle V., Yeo S.-H., Colledge W. H. (2013). Does kisspeptin signaling have a role in the testes?. *Frontiers in Endocrinology*.

[26] Irfan S., Ehmcke J., Shahab M., Wistuba J., Schlatt S. (2016). Immunocytochemical localization of kisspeptin and kisspeptin receptor in the primate testis. *Journal of Medical Primatology*.

[27] Yoshida M., Yoshida K. (2011). Sperm chemotaxis and regulation of flagellar movement by Ca2+. *Molecular Human Reproduction*.

[28] Teves M. E., Barbano F., Guidobaldi H. A., Sanchez R., Miska W., Giojalas L. C. (2006). Progesterone at the picomolar range is a chemoattractant for mammalian spermatozoa. *Fertility and Sterility*.

[29] Fukuda N., Yomogida K., Okabe M., Touhara K. (2004). Functional characterization of a mouse testicular olfactory receptor and its role in chemosensing and in regulation of sperm motility. *Journal of Cell Science*.

[30] Liu X., Lee K., Herbison A. E. (2008). Kisspeptin excites gonadotropin-releasing hormone neurons through a phospholipase C/calcium-dependent pathway regulating multiple ion channels. *Endocrinology*.

