# Supplementary material for: Kisspeptin Protein in Seminal Plasma Is Positively Associated with Semen Quality: Results from the MARHCS Study in Chongqing, China
Source: Biomed Res Int. 2019 Jan 9;2019:5129263. doi: 10.1155/2019/5129263 (PMC6343164; doi:10.1155/2019/5129263)
Supplement: Supplementary Materials — Figure: correlation between kisspeptin in semen plasma and kisspeptin in blood plasma. There was no statistically significant association between kisspeptin in semen plasma and kisspeptin in blood plasma. [file 5129263.f1.pdf]

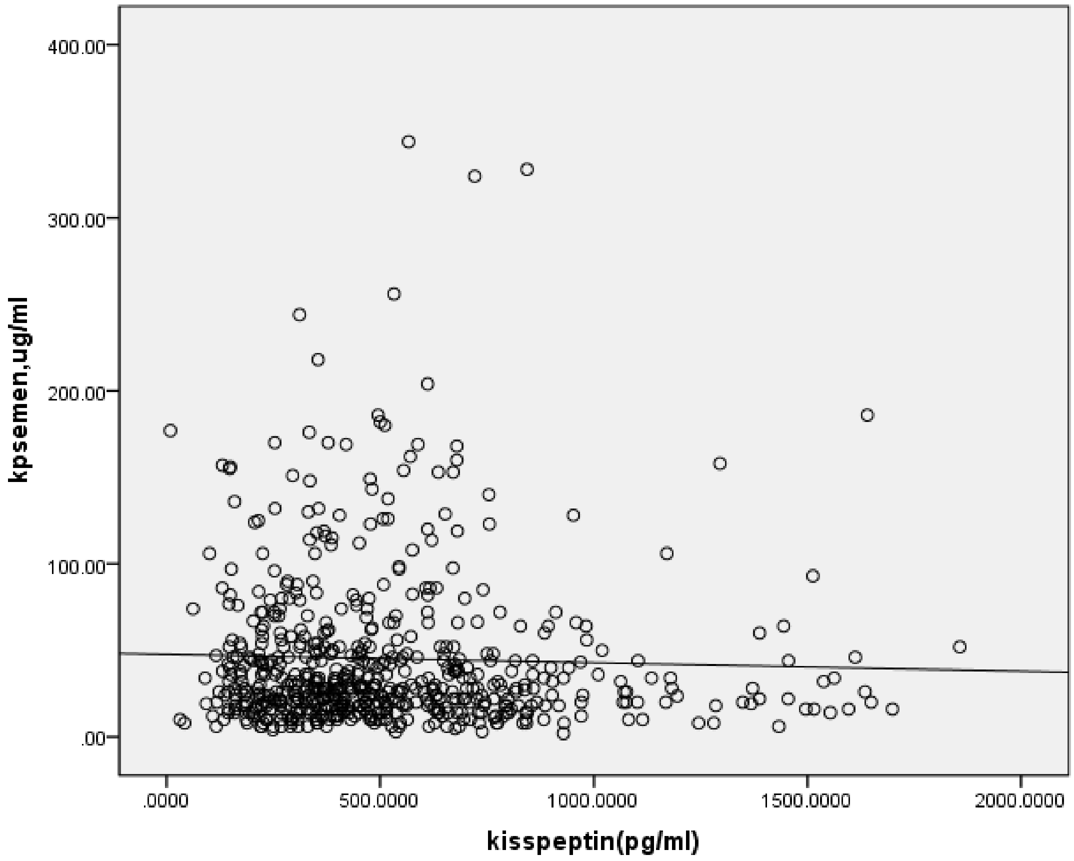

Supplemental figure. Scatter plot of kisspeptin in semen plasma versus kisspeptin in blood plasma. There was no statistically significant association between kisspeptin in semen plasma and kisspeptin in blood plasma.
